# Supplementary material for: Co-circulation of multiple influenza A reassortants in swine harboring genes from seasonal human and swine influenza viruses
Source: eLife. 2021 Jul 27;10:e60940. doi: 10.7554/eLife.60940 (PMC8397370; doi:10.7554/eLife.60940)

Figure 4 - figure supplement 3. Bayesian phylogenetic tree of the NS sequences of the Danish swIAV surveillance 2011-2018. A/sw/Denmark/12687/2003(H1N2) accession number: KC900267 was used as an outgroup. A blue taxon indicates that the NS gene of the sample is of avian-like origin, whereas a black taxon indicates that the NS gene of the sample is of H1N1pmd09 origin. Sequences are named according to their sequence ID and lineage.


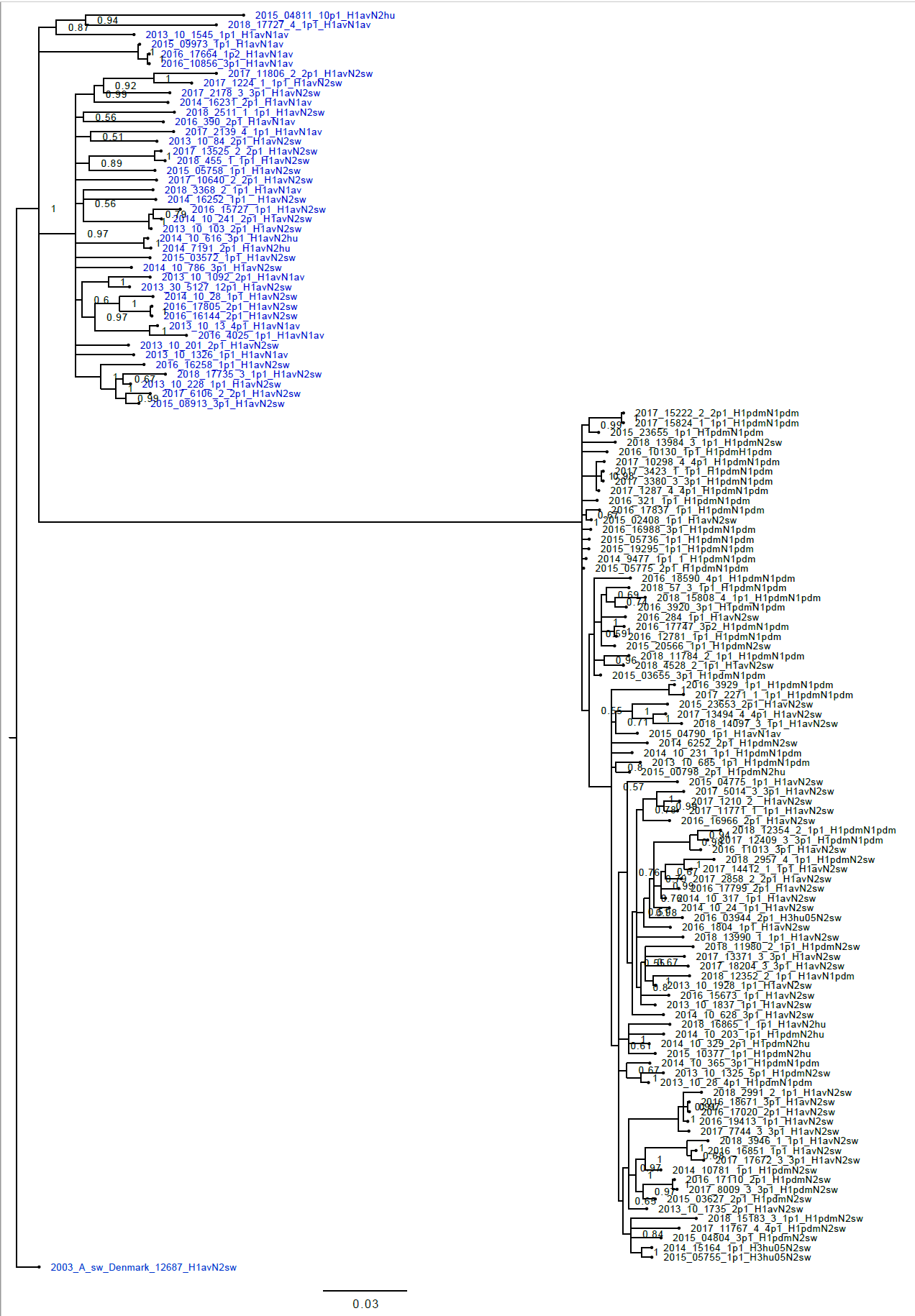

Supplement: Table 4—source data 3. — A/sw/Denmark/12687/2003(H1N2) accession number: KC900267 was used as an outgroup. A blue taxon indicates that the NS gene of the sample is of avian-like origin, whereas a black taxon indicates that the NS gene of the sample is of H1N1pmd09 origin. Sequences are named according to their sequence ID and lineage. [file elife-60940-table4-data3.docx]
