# Supplementary material for: Co-circulation of multiple influenza A reassortants in swine harboring genes from seasonal human and swine influenza viruses
Source: eLife. 2021 Jul 27;10:e60940. doi: 10.7554/eLife.60940 (PMC8397370; doi:10.7554/eLife.60940)

Figure 4 - figure supplement 7. Percentage of H1N2dk isolates containing at least one internal gene of H1N1pdm09 origin.


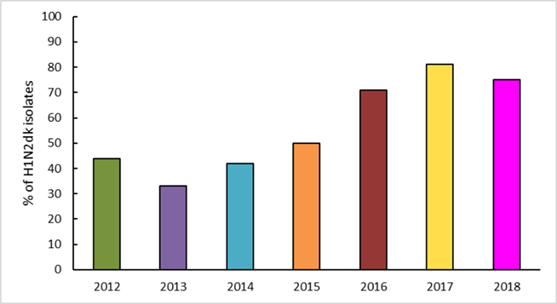

Supplement: Table 4—source data 7. [file elife-60940-table4-data7.docx]
