# Supplementary material for: Co-circulation of multiple influenza A reassortants in swine harboring genes from seasonal human and swine influenza viruses
Source: eLife. 2021 Jul 27;10:e60940. doi: 10.7554/eLife.60940 (PMC8397370; doi:10.7554/eLife.60940)
Supplement: Supplementary file 1. [file elife-60940-supp1.docx]

Supplementary File 1. The genotype of all full genome sequenced samples during the Danish swIAV surveillance 2011-2018.

|  | | | | HA | NA | M | NP | NS | PA | PB1 | PB2 | Accession no. |
| --- | --- | --- | --- | --- | --- | --- | --- | --- | --- | --- | --- | --- |
| H1N2dk | | | | | | | | | | | |  |
| A/Swine/Denmark/2013-10-84-2p1/2013(H1N2) | | | |  |  |  |  |  |  |  |  | MT666504-11 |
| A/Swine/Denmark/2013-10-103-2p1/2013(H1N2) | | | |  |  |  |  |  |  |  |  | MT666441-47 |
| A/Swine/Denmark/2013-10-201-2p1/2013(H1N2) | | | |  |  |  |  |  |  |  |  | MT666821-28 |
| A/Swine/Denmark/2013-10-228-1p1/2013(H1N2) | | | |  |  |  |  |  |  |  |  | MT666356-62 |
| A/Swine/Denmark/2013-30-5127-12p1/2013(H1N2) | | | |  |  |  |  |  |  |  |  | MT666789-96 |
| A/Swine/Denmark/2013-10-1735-2p1/2013(H1N2) | | | |  |  |  |  |  |  |  |  | MT666757-64 |
| A/Swine/Denmark/2013-10-1837-1p1/2013(H1N2) | | | |  |  |  |  |  |  |  |  | MT666694-01 |
| A/Swine/Denmark/2013-10-1928-1p1/2013(H1N2) | | | |  |  |  |  |  |  |  |  | MT666702-09 |
| A/Swine/Denmark/2014-10-24-1p1/2014(H1N2) | | | |  |  |  |  |  |  |  |  | MT666734-41 |
| A/Swine/Denmark/2014-10-28-1p1/2014(H1N2) | | | |  |  |  |  |  |  |  |  | MT666480-87 |
| A/Swine/Denmark/2014-10-241-1p1/2014(H1N2) | | | |  |  |  |  |  |  |  |  | MT666433-40 |
| A/Swine/Denmark/2014-10-317-1p1/2014(H1N2) | | | |  |  |  |  |  |  |  |  | MT666742-49 |
| A/Swine/Denmark/2014-10-628-3p1/2014(H1N2) | | | |  |  |  |  |  |  |  |  | MT666662-69 |
| A/Swine/Denmark/2014-10-786-3p1/2014(H1N2) | | | |  |  |  |  |  |  |  |  | MT666280-87 |
| A/Swine/Denmark/2014-16252-1p1/2014(H1N2) | | | |  |  |  |  |  |  |  |  | MT666418-25 |
| A/Swine/Denmark/2015-02408-1p1/2015(H1N2) | | | |  |  |  |  |  |  |  |  | MT666686-93 |
| A/Swine/Denmark/2015-03572-1p1/2015(H1N2) | | | |  |  |  |  |  |  |  |  | MT666272-79 |
| A/Swine/Denmark/2015-04775-1p1/2015(H1N2) | | | |  |  |  |  |  |  |  |  | MT666248-55 |
| A/Swine/Denmark/2015-05758-1p1/2015(H1N2) | | | |  |  |  |  |  |  |  |  | MT666472-79 |
| A/Swine/Denmark/2015-08913-3p1/2015(H1N2) | | | |  |  |  |  |  |  |  |  | MT666363-70 |
| A/Swine/Denmark/2015-23653-2p1/2015(H1N2) | | | |  |  |  |  |  |  |  |  | MT666710-17 |
| A/Swine/Denmark/2016-284-1p1/2016(H1N2) | | | |  |  |  |  |  |  |  |  | MT666348-55 |
| A/Swine/Denmark/2016-1804-1p1/2016(H1N2) | | | |  |  |  |  |  |  |  |  | MT666638-45 |
| A/Swine/Denmark/2016-11013-3p1/2016(H1N2) | | | |  |  |  |  |  |  |  |  | MT666630-37 |
| A/Swine/Denmark/2016-15673-1p1/2016(H1N2) | | | |  |  |  |  |  |  |  |  | MT666583-90 |
| A/Swine/Denmark/2016-15727-1p1/2016(H1N2) | | | |  |  |  |  |  |  |  |  | MT666426-32 |
| A/Swine/Denmark/2016-16144-2p1/2016(H1N2) | | | |  |  |  |  |  |  |  |  | MT666488-95 |
| A/Swine/Denmark/2016-16258-1p1/2016(H1N2) | | | |  |  |  |  |  |  |  |  | MT666386-93 |
| A/Swine/Denmark/2016-16851-1p1/2016(H1N2) | | | |  |  |  |  |  |  |  |  | MT666567-74 |
| A/Swine/Denmark/2016-16966-2p1/2016(H1N2) | | | |  |  |  |  |  |  |  |  | MT666591-97 |
| A/Swine/Denmark/2016-17020-2p1/2016(H1N2) | | | |  |  |  |  |  |  |  |  | MT666319-25 |
| A/Swine/Denmark/2016-17799-2p1/2016(H1N2) | | | |  |  |  |  |  |  |  |  | MT666718-25 |
| A/Swine/Denmark/2016-17805-2p1/2016(H1N2) | | | |  |  |  |  |  |  |  |  | MT666496-03 |
| A/Swine/Denmark/2016-18671-3p1/2016(H1N2) | | | |  |  |  |  |  |  |  |  | MT666312-18 |
| A/Swine/Denmark/2016-19413-1p1/2016(H1N2) | | | |  |  |  |  |  |  |  |  | MT666326-32 |
| A/Swine/Denmark/2017-1224-1p1/2017(H1N2) | | | |  |  |  |  |  |  |  |  | MT666378-85 |
| A/Swine/Denmark/2017-1210-2p1/2017(H1N2) | | | |  |  |  |  |  |  |  |  | MT666551-58 |
| A/Swine/Denmark/2017-2178-3p1/2017(H1N2) | | | |  |  |  |  |  |  |  |  | MT666288-95 |
| A/Swine/Denmark/2017-2858-2p1/2017(H1N2) | | | |  |  |  |  |  |  |  |  | MT666750-56 |
| A/Swine/Denmark/2017-5014-3p1/2017(H1N2) | | | |  |  |  |  |  |  |  |  | MT666614-21 |
| A/Swine/Denmark/2017-6106-2p1/2017(H1N2) | | | |  |  |  |  |  |  |  |  | MT666371-77 |
| A/Swine/Denmark/2017-7744-3p1/2017(H1N2) | | | |  |  |  |  |  |  |  |  | MT666654-61 |
| A/Swine/Denmark/2017-10640-2p1/2017(H1N2) | | | |  |  |  |  |  |  |  |  | MT666606-13 |
| A/Swine/Denmark/2017-11771-1p1/2017(H1N2) | | | |  |  |  |  |  |  |  |  | MT666543-50 |
| A/Swine/Denmark/2017-11806-2p1/2017(H1N2) | | | |  |  |  |  |  |  |  |  | MT666394-01 |
| A/Swine/Denmark/2017-13371-3p1/2017(H1N2) | | | |  |  |  |  |  |  |  |  | MT666670-77 |
| A/Swine/Denmark/2017-13494-4p1/2017(H1N2) | | | |  |  |  |  |  |  |  |  | MT666678-85 |
| A/Swine/Denmark/2017-13525-2p1/2017(H1N2) | | | |  |  |  |  |  |  |  |  | MT666464-71 |
| A/Swine/Denmark/2017-14412-1p1/2017(H1N2) | | | |  |  |  |  |  |  |  |  | MT666726-33 |
| A/Swine/Denmark/2017-17672-3p1/2017(H1N2) | | | |  |  |  |  |  |  |  |  | MT666575-82 |
| A/Swine/Denmark/2017-18204-3p1/2017(H1N2) | | | |  |  |  |  |  |  |  |  | MT666536-42 |
| A/Swine/Denmark/2018-455-1-1p1/2018(H1N2) | | | |  |  |  |  |  |  |  |  | MT666528-35 |
| A/Swine/Denmark/2018-2511-1-1p1/2018(H1N2) | | | |  |  |  |  |  |  |  |  | MT666456-63 |
| A/Swine/Denmark/2018-2991-2-1p1/2018(H1N2) | | | |  |  |  |  |  |  |  |  | MT666304-11 |
| A/Swine/Denmark/2018-3946-1-1p1/2018(H1N2) | | | |  |  |  |  |  |  |  |  | MT666559-66 |
| A/Swine/Denmark/2018-4528-2-1p1/2018(H1N2) | | | |  |  |  |  |  |  |  |  | MT666512-19 |
| A/Swine/Denmark/2018-13990-1-1p1/2018(H1N2) | | | |  |  |  |  |  |  |  |  | MT666520-27 |
| A/Swine/Denmark/2018-14097-3-1p1/2018(H1N2) | | | |  |  |  |  |  |  |  |  | MT666598-05 |
| A/Swine/Denmark/2018-17735-3-1p1/2018(H1N2) | | | |  |  |  |  |  |  |  |  | MT666448-55 |
| H1avN1av | | | | | | | | | | | |  |
| A/Swine/Denmark/2013-10-13-4p1/2013(H1N1) | | | |  |  |  |  |  |  |  |  | MT666805-12 |
| A/Swine/Denmark/2013-10-1092-2p1/2013(H1N1) | | | |  |  |  |  |  |  |  |  | MT666781-88 |
| A/Swine/Denmark/2013-10-1326-1p1/2013(H1N1) | | | |  |  |  |  |  |  |  |  | MT666296-03 |
| A/Swine/Denmark/2013-10-1545-1p1/2013(H1N1) | | | |  |  |  |  |  |  |  |  | MT666813-20 |
| A/Swine/Denmark/2014-16231-3p1/2014(H1N1) | | | |  |  |  |  |  |  |  |  | MT666341-47 |
| A/Swine/Denmark/2015-4790-1p1 /2015(H1N1) | | | |  |  |  |  |  |  |  |  | MT666333-40 |
| A/Swine/Denmark/2015-09973-1p1/2015(H1N1) | | | |  |  |  |  |  |  |  |  | MT666225-32 |
| A/Swine/Denmark/2016-390-2p1/2016(H1N1) | | | |  |  |  |  |  |  |  |  | MT666264-71 |
| A/Swine/Denmark/2016-4025-1p1/2016(H1N1) | | | |  |  |  |  |  |  |  |  | MT666797-04 |
| A/Swine/Denmark/2016-10856-3p1/2016(H1N1) | | | |  |  |  |  |  |  |  |  | MT666240-47 |
| A/Swine/Denmark/2016-17664-1p2/2016(H1N1) | | | |  |  |  |  |  |  |  |  | MT666233-39 |
| A/Swine/Denmark/2017-2139-4/2017(H1N1) | | | |  |  |  |  |  |  |  |  | MT666256-63 |
| A/Swine/Denmark/2018-3368-2-1p1/2018(H1N1) | | | |  |  |  |  |  |  |  |  | MT666410-17 |
| A/Swine/Denmark/2018-17727-4-1p1/2018(H1N1) | | | |  |  |  |  |  |  |  |  | MT666402-09 |
| H1avN2hu95 | | | | | | | | | | | |  |
| A/Swine/Denmark/2014-10-616-3p1/2014(H1N2) | | | |  |  |  |  |  |  |  |  | MT666765-72 |
| A/Swine/Denmark/2014-7191-2p1/2014(H1N2) | | | |  |  |  |  |  |  |  |  | MT666773-80 |
| A/Swine/Denmark/2015-04811-10p1/2015(H1N2) | | | |  |  |  |  |  |  |  |  | MT666829-36 |
| A/Swine/Denmark/2018-16865-1-1p1/2018(H1N2) | | | |  |  |  |  |  |  |  |  | MT666646-53 |
| H1N1pdm09 | | | | | | | | | | | |  |
| A/Swine/Denmark/2013-10-28-4p1/2013(H1N1) | | | |  |  |  |  |  |  |  |  | MT667172-78 |
| A/Swine/Denmark/2013-10-685-1p1/2013(H1N1) | | | |  |  |  |  |  |  |  |  | MT667203-09 |
| A/Swine/Denmark/2014-10-231-1p1/2014(H1N1) | | | |  |  |  |  |  |  |  |  | MT667210-17 |
| A/Swine/Denmark/2014-10-365-3p1/2014(H1N1) | | | |  |  |  |  |  |  |  |  | MT667156-63 |
| A/Swine/Denmark/2014-9477-1p1/2014(H1N1) | | | |  |  |  |  |  |  |  |  | MT667060-67 |
| A/Swine/Denmark/2015-03655-3p1/2015(H1N1) | | | |  |  |  |  |  |  |  |  | MT666996-03 |
| A/Swine/Denmark/2015-05736-1p1/2015(H1N1) | | | |  |  |  |  |  |  |  |  | MT667068-75 |
| A/Swine/Denmark/2015-05775-2p1/2015(H1N1) | | | |  |  |  |  |  |  |  |  | MT667036-43 |
| A/Swine/Denmark/2015-19295-1p1/2015(H1N1) | | | |  |  |  |  |  |  |  |  | MT667044-51 |
| A/Swine/Denmark/2015-23655-1p1/2015(H1N1) | | | |  |  |  |  |  |  |  |  | MT667004-11 |
| A/Swine/Denmark/2016-321-1p1/2016(H1N1) | | | |  |  |  |  |  |  |  |  | MT667052-59 |
| A/Swine/Denmark/2016-3920-3p1/2016(H1N1) | | | |  |  |  |  |  |  |  |  | MT666957-64 |
| A/Swine/Denmark/2016-3929-1p1/2016(H1N1) | | | |  |  |  |  |  |  |  |  | MT667187-94 |
| A/Swine/Denmark/2016-10130-1p1/2016(H1N1) | | | |  |  |  |  |  |  |  |  | MT666988-95 |
| A/Swine/Denmark/2016-12781-1p1/2016(H1N1) | | | |  |  |  |  |  |  |  |  | MT666933-40 |
| A/Swine/Denmark/2016-16988-3p1/2016(H1N1) | | | |  |  |  |  |  |  |  |  | MT666980-87 |
| A/Swine/Denmark/2016-17747-3p2/2016(H1N1) | | | |  |  |  |  |  |  |  |  | MT666941-48 |
| A/Swine/Denmark/2016-17837-1p1/2016(H1N1) | | | |  |  |  |  |  |  |  |  | MT667028-35 |
| A/Swine/Denmark/2016-18590-4p1/2016(H1N1) | | | |  |  |  |  |  |  |  |  | MT666885-92 |
| A/Swine/Denmark/2017-1287-4p1/2017(H1N1) | | | |  |  |  |  |  |  |  |  | MT666909-16 |
| A/Swine/Denmark/2017-2271-1p1/2017(H1N1) | | | |  |  |  |  |  |  |  |  | MT667179-86 |
| A/Swine/Denmark/2017-3380-3p1/2017(H1N1) | | | |  |  |  |  |  |  |  |  | MT666917-24 |
| A/Swine/Denmark/2017-3423-1p1/2017(H1N1) | | | |  |  |  |  |  |  |  |  | MT666925-32 |
| A/Swine/Denmark/2017-10298-4p1/2017(H1N1) | | | |  |  |  |  |  |  |  |  | MT666901-08 |
| A/Swine/Denmark/2017-12409-3p1/2017(H1N1) | | | |  |  |  |  |  |  |  |  | MT666965-71 |
| A/Swine/Denmark/2017-15222-2p1/2017(H1N1) | | | |  |  |  |  |  |  |  |  | MT667012-19 |
| A/Swine/Denmark/2017-15824-1p1/2017(H1N1) | | | |  |  |  |  |  |  |  |  | MT666845-52 |
| A/Swine/Denmark/2018-57-3-1p1/2018(H1N1) | | | |  |  |  |  |  |  |  |  | MT666853-60 |
| A/Swine/Denmark/2018-11784-2-1p1/2018(H1N1) | | | |  |  |  |  |  |  |  |  | MT666877-84 |
| A/Swine/Denmark/2018-12354-2-1p1/2018(H1N1) | | | |  |  |  |  |  |  |  |  | MT666972-79 |
| A/Swine/Denmark/2018-15808-4-1p1/2018(H1N1) | | | |  |  |  |  |  |  |  |  | MT666845-52 |
| H1pdmN2dk | | | | | | | | | | | |  |
| A/Swine/Denmark/2013-10-1325-5p1/2013(H1N2) | | | |  |  |  |  |  |  |  |  | MT667164-71 |
| A/Swine/Denmark/2014-6252-2p1/2014(H1N2) | | | |  |  |  |  |  |  |  |  | MT667195-02 |
| A/Swine/Denmark/2014-10781-1p1/2014(H1N2) | | | |  |  |  |  |  |  |  |  | MT667108-15 |
| A/Swine/Denmark/2015-03627-2p1/2015(H1N2) | | | |  |  |  |  |  |  |  |  | MT667116-23 |
| A/Swine/Denmark/2015-04804-3p1/2015(H1N2) | | | |  |  |  |  |  |  |  |  | MT667148-55 |
| A/Swine/Denmark/2015-20566-1p1/2015(H1N2) | | | |  |  |  |  |  |  |  |  | MT666893-00 |
| A/Swine/Denmark/2016-17110-2p1/2016(H1N2) | | | |  |  |  |  |  |  |  |  | MT667124-31 |
| A/Swine/Denmark/2017-8009-3p1/2017(H1N2) | | | |  |  |  |  |  |  |  |  | MT667132-39 |
| A/Swine/Denmark/2017-11767-4p1/2017(H1N2) | | | |  |  |  |  |  |  |  |  | MT667076-83 |
| A/Swine/Denmark/2018-2957-4-1p1/2018(H1N2) | | | |  |  |  |  |  |  |  |  | MT666949-56 |
| A/Swine/Denmark/2018-11980-2-1p1/2018(H1N2) | | | |  |  |  |  |  |  |  |  | MT666869-76 |
| A/Swine/Denmark/2018-13984-3-1p1/2018(H1N2) | | | |  |  |  |  |  |  |  |  | MT666861-68 |
| A/Swine/Denmark/2018-15183-3-1p1/2018(H1N2) | | | |  |  |  |  |  |  |  |  | MT666837-44 |
| H1pdmN2hu95 | | | | | | | | | | | |  |
| A/Swine/Denmark/2014-10-203-1p1/2014(H1N2) | | | |  |  |  |  |  |  |  |  | MT667092-99 |
| A/Swine/Denmark/2014-10-329-2p1/2014(H1N2) | | | |  |  |  |  |  |  |  |  | MT667100-07 |
| A/Swine/Denmark/2015-00798-2p1/2015(H1N2) | | | |  |  |  |  |  |  |  |  | MT667140-47 |
| A/Swine/Denmark/2015-10377-1p1/2015(H1N2) | | | |  |  |  |  |  |  |  |  | MT667084-91 |
| H3hu05N2dk | | | | | | | | | | | |  |
| A/Swine/Denmark/2014-15164-1p1/2014(H3N2) | | | |  |  |  |  |  |  |  |  | EPI-ISL-247092 |
| A/Swine/Denmark/2015-05755-1p1/2015(H3N2) | | | |  |  |  |  |  |  |  |  | MT667226-33 |
| A/Swine/Denmark/2016-3944-2p1/2016(H3N2) | | | |  |  |  |  |  |  |  |  | MT667218-25 |
| H1avN1pdm | | | | | | | | | | | |  |
| A/Swine/Denmark/2018-12352-2-1p1/2018(H1N1) | | | |  |  |  |  |  |  |  |  | MT666622-29 |
| Color codes | | | | | | | | | | | |  |
|  |  |  |  | Enzootic swine origin ( H1avN1av, H1N2dk, H3N2) | | | | | H1N1pdm09 origin | | |  |
|  |  |  |  | Seasonal human H3N2 origin | | | | | Not able to sequence | | |  |
