# Supplementary material for: Co-circulation of multiple influenza A reassortants in swine harboring genes from seasonal human and swine influenza viruses
Source: eLife. 2021 Jul 27;10:e60940. doi: 10.7554/eLife.60940 (PMC8397370; doi:10.7554/eLife.60940)
Supplement: Supplementary file 2. — Aa positions are numbered according to the first methionine if nothing else is indicated. [file elife-60940-supp2.docx]

Supplementary File 2. Residues examined for specific mutations involving host adaptation, virulence, pathogenicity and dominating residues differing between H1av and H1N1pdm09 origin viruses. Aa positions are numbered according to the first methionine if nothing else is indicated.

| Protein and residue/aa change | Function | H1av-origin | H1N1pdm09 origin |
| --- | --- | --- | --- |
| PB2 |  |  |  |
| T271A | Dominant residue differ between avian- and H1N1pdm09 viruses^59^ | 35/36 had T  1/36 had I | 85/85 had A |
| E627K | Increased viral replication and virulence^84^ | 0/78 | 0/48 |
| D701N | Enhance viral replication and pathogenicity of Eurasian avian like swIAV^85^ | 36/78 | 0/48 |
| K702R | Host-adaption; at this residue avian influenza carries lysine while most mammalian IAV carry an arginine^86^ | 0/78 | 0/48 |
| PB1 |  |  |  |
| V336I | Dominant residue differ between avian- and H1N1pdm09 viruses^59^ | 34/37 had V  3/37 had I | 87/87 had I |
| PA |  |  |  |
| K356R | As above^59^ | 38/38 had K | 88/91 had R |
| S409N | As above^59^ | 5/38 had S  33/38 had N | 90/91 had N  1/91 had S |
| HA |  |  |  |
| T200A (H3 numbering) | Increased receptor binding affinity to the α2.6-linked sialic acid for H1N1pdm09 viruses^79^ | 1/78 had A | 48/48 had A |
| E190D (H3 numbering) | change in receptor specificity of avian IAV from α2.3-linked sialic acid to α2.6-linked sialic acid^78^ | 73/78  5/78 had V or S | 40/48  8/48 had S or G (all Hu-L) |
| G225D (H3 numbering) | As above^78^ | 6/78  63/78 had E  9/78 had T/K/N | 18/48 (14/18 was Hu-L)  23/48 had N  1/48 had G |
| K159, G172E, I183V, S200P, S202N, D204S, V338I and V66I | Related to the H1pdmN2sw lineage in Germany^27^ |  | Not specific for H1pdmN2sw. However,  I173V and V466I were present in 3/12 and 1/12 of the Danish H1pdmN2sw viruses |
| NP |  |  |  |
| K48Q | Confer MxA resistance in combination with R98K and R99K for the NP protein of avian-like swine origin^60^ | 35/35 had Q | 92/92 had K |
| E53D | Confer MxA resistance for the NP protein of H1N1pdm09 origin^61^ | 35/35 had E | 10/92 had D (8/10 were H1pdmNx viruses belonging to the Hu-L cluster) |
| R98K | Confer MxA resistance in combination with K48Q and R99K for the NP protein of avian-like swine origin^60^ | 33/35 had K | 92/92 had R |
| R99K | Confer MxA resistance in combination with K48Q and R98K for the NP protein of avian-like swine origin^60^ | 31/35 had K | 90/92 had R |
| R100I/V | Confer MxA resistance for the NP protein of H1N1pdm09 origin^61^ and dominant residue differ between avian- and H1N1pdm09 viruses^59^ | 35/35 had R | 84/92 had I  3/92 had M  3/92 had V  2/92 T/L |
| F313V | Confer MxA resistance for the NP protein of H1N1pdm09 origin^61^ | 35/35 had F | 91/92 had V |
| V33I | Dominant residue differ between avian- and H1N1pdm09 viruses^59^ | 8/35 had V  27/35 had I | 91/92 had I  1/92 had V |
| R100V | As above^59^ |  |  |
| R305K | As above^59^ | 35/35 had R | 91/92 had K  1/92 had R |
| Q357K | As above^59^ | 35/35 had Q | 92/92 had K |
