# Supplementary material for: Co-circulation of multiple influenza A reassortants in swine harboring genes from seasonal human and swine influenza viruses
Source: eLife. 2021 Jul 27;10:e60940. doi: 10.7554/eLife.60940 (PMC8397370; doi:10.7554/eLife.60940)
Supplement: Supplementary file 3. — Aa positions are numbered according to the first methionine. [file elife-60940-supp3.docx]

Supplementary File 3. Amino acid differences in the internal proteins of the Danish sequences of the Sw-L cluster and the sequences located outside the cluster. Aa positions are numbered according to the first methionine.

| Protein | Aa change Hu-L → Sw-L | Prevalence in the sequences located outside the Sw-L cluster | Prevalence in the sequences of the Sw-L cluster |
| --- | --- | --- | --- |
| PB2 | T76A | 1/18 | 22/30 |
|  | M283I | 0 | 27/30 |
|  | V359M | 6/18 | 27/30 |
|  | N456S | 4/18 | 25/30 |
| PB1 | T110A | 2/16 | 27/30 |
|  | M317I | 3/16 | 29/30 |
| NS | V117M | 0/18 | 24/30 |
| PA | C241Y | 5/18 | 28/30 |
| NP | R212L | 0/18 | 27/30 |
