# Supplementary material for: Co-circulation of multiple influenza A reassortants in swine harboring genes from seasonal human and swine influenza viruses
Source: eLife. 2021 Jul 27;10:e60940. doi: 10.7554/eLife.60940 (PMC8397370; doi:10.7554/eLife.60940)
Supplement: Supplementary file 4. — Nucleotides are named according to the IUPAC codes. Purple color indicates the pQE part of the primers. [file elife-60940-supp4.docx]

Supplementary File 4. Primers and probes used for detection, subtyping and full genome sequencing of swIAV. Nucleotides are named according to the IUPAC codes. Purple color indicate the pQE part of the primers.

|  | | Primer/probe | Sequence (5’ – 3’) | Ref. |
| --- | --- | --- | --- | --- |
| **Detection** | M | Rim-F | CTTCTAACCGAGGTCGAAACG | ^71^ |
|  |  | Rim-R | FAM-AGGGCATTTTGGACAAAKCGTCTA |  |
|  |  | MaProbe | CCCAGTGAGCGAGGACTGCAGCGT |  |
| **A(H1N1)pdm09** | H1pdm(sw) | H1fw2sw-3 | GAAGTTCAAGCCGGAAATAGCA | ^73^ |
|  |  | H1rev2sw-2 | CCC GGC TCT ACT AGT GTC CA |  |
|  |  | H1probe2sw-3 | AT488-CCC AAA GTG AGG RAT CAA GAA GGG AG-BHQ1 |  |
|  | H1pdm(hu) | H1pdm_Fw  H1pdm_Rv  H1pdm_P | CTAGTGGTACCGAGATATGCA  TATTGCAATCGTGGACTGGTGT  FAM-CGCAATGGAAAGAAATGCTGGATCTGG-BHQ1 | In house |

|  | H1av | H1-av-F | GAA GGR GGA TGG ACA GGA ATG A | ^34,73^ |
| --- | --- | --- | --- | --- |
|  |  | H1-av-R | CAA TTA HTG ART TCA CTT TGT TGC TG |  |
|  |  | H1-av-P | ROX-TCT GGT TAC GCA GCW GAT CAG AAA AGC AC- BHQ2 |  |
| Subtyping | H3sw | H3-sw-F | TGA TGG AGC AAA TTG CAC ACT G | ^34,73^ |
|  |  | H3-sw-R | CGT TCA ATG AAA AGG TCC CAT TTC |  |
|  |  | H3-sw-P | AT680-CAC AAT GAG GGT CCC CTA ATA GAG CGT CCA-BBQ |  |
|  | H3hu05 | H3-hu-F | GATGA TGG AGA AAA CTG CAC ACT A | ^34,73^ |
|  |  | H3-hu-R | CGT TCA ACA AAA AGG TCC CAT TTC |  |
|  |  | H3-hu-P | AT680-CAC ACT GAG GGT CTC CCA ATA GAG CAT CTA-BBQ |  |
|  | N1av | N1-F | CCTTGCTTCTGGGTTGAACTAATC | ^34,73^ |
|  |  | N1-R | AGTGTCACTATTTACACCACAAAAGG |  |
|  |  | N1-P | ROX-TGCTCCCGCTAGTCCAGATTGTGTTCTCTT-BHQ2 |  |
|  | N1pdm | N1pdm-F | CGAAATGAGTGCCCCTAATTATC | ^34,73^ |
|  |  | N1pdm-R | CGATTCGAGCCATGCCAGTTA |  |
|  |  | N1pdm-P | FAM-[+C][+C]T[+G]ATTCT[+A]GTGAAATCA[+C]-BHQ1 |  |
|  | N2dk | N2-F | GAGTATGGTGGACBTCAAAYAG | ^34,73^ |
|  |  | N2-R | TTGCGAAAGCTTATATAGGCATGA |  |
|  |  | N2-P | AF532-CCA TCA GGC CAT GAG CCT GAV CCA TA-BHQ1 |  |
|  | N2hu95 | N2hu-F | CTGGTATTTTCTCTGTTGAAGGC | ^34,73^ |
|  |  | N2hu-R | CCA SAC TTC AKT TTC CTG YTT CC |  |
|  |  | N2hu-P | AF532-T[+C]A [+A]CT CYA CAT AAA AGC ACC [+G]-BHQ1 |  |
|  | NS | H3NSF1 | AGC AAA AGC AGG GTG ACA AAG ACA | In house |
|  |  | H3NSR1 | AGT AGA AAC AAG GGT GTT TTT TAT |  |
|  | M | MF8 | GCA GGT AGA TAT TGA AAG ATG AG | In house |
| Full length RT-PCR |  | MR1025 | AGA AAC AAG GTA GTT TTT TAC TC |  |
|  | NA | pQE-NA-F | CGGATAACAATTTCACACAGAGCAAAAGCAGGAGT | In house |
|  |  | pQE-NA-R | GTTCTGAGGTCATTACTGGAGTAGAAACAAGGAGTTTTTT |  |
|  | NP | pQE-NP-F2 | CGGATAACAATTTCACACAGAGCAAAAGCAGGGTAGATAATC | In house |
|  |  | pQE-NP-R | GTTCTGAGGTCATTACTGGAGTAGAAACAAGGGTATTTTT |  |
|  | HA | pQE-HAs-F | CGGATAACAATTTCACACAGAGCAAAAGCAGGGGAWAATW | In house |
|  |  | pQE-HA-R | GTTCTGAGGTCATTACTGGAGTAGAAACAAGGGTGTTTT |  |
|  | H1pdm | pQE-HApd-F | CGGATAACAATTTCACACAGAGCAAAAGCAGGGGAAAAC | In house |
|  |  | pQE-HA-R | GTTCTGAGGTCATTACTGGAGTAGAAACAAGGGTGTTTT |  |
|  | PA | pQE-PA-F2 | CGGATAACAATTTCACACAGAGCAAAAGCAGGTAC | In house |
|  |  | pQE-PA-R | GTTCTGAGGTCATTACTGGAGTAGAAACAAGGTACTT |  |
|  | PB1 | pQE-PB1-F2 | CGGATAACAATTTCACACAGAGCRAAAGCAGGCAAAC | In house |
|  |  | pQE-PB1-R | GTTCTGAGGTCATTACTGGAGTAGAAACAAGGCATTT |  |
|  | PB2 | pQE-PB2-F3 | CGGATAACAATTTCACACAGAGCRAAAGCAGGTCAAAT | In house |
|  |  | pQE-PB2-R | GTTCTGAGGTCATTACTGGAGTAGAAACAAGGTCGTTT |  |
|  |  |  |  |  |
|  | Universal | MBTuni-12 | ACGCGTGATCAGCRAAAGCAGG | ^74^ |
|  | IAV primers | MBTuni-13 | ACGCGTGATCAGTAGAAACAAGG |  |
